# Diploma Mills

**Published:** 1881-05

**Authors:** 


Diploma Mills.—"Dean" Buchanan, it seems, has recently confessed to a knowledge of the existence of twenty-five bogus "diploma mills," and the distribution of 20,000 bogus diplomas in this country, and 40,000 in Europe. We are glad the old rascal has been brought to make so much of a confession; but does he tell all? It may be he feels some contrition for his sins, now that he is incarcerated within prison walls; but were he at large, his nefarious traffic would probably be resumed again. "When the devil fell sick, the devil a monk would be; but when the devil got well, the devil a monk was he."

